# Analysing the effects of distance, taxon and biomass on vertebrate detections using bulk-collected carrion fly iDNA

**DOI:** 10.1098/rsos.231286

**Published:** 2024-04-03

**Authors:** Kristen Fernandes, Philip W. Bateman, Benjamin J. Saunders, Mark Gibberd, Michael Bunce, Kristine Bohmann, Paul Nevill

**Affiliations:** ^1^ Trace and Environmental DNA (TrEnD) Laboratory, School of Molecular and Life Sciences, Curtin University, Bentley, Western Australia 6102, Australia; ^2^ Section for Molecular Ecology and Evolution, Faculty of Health and Medical Sciences, Globe Institute, University of Copenhagen, Copenhagen, Denmark; ^3^ Food Agility CRC Ltd, Sydney, New South Wales 2000, Australia; ^4^ Department of Anatomy, University of Otago, Dunedin 9016, New Zealand; ^5^ Behavioural Ecology Laboratory, School of Molecular and Life Sciences, Curtin University, Bentley, Western Australia 6102, Australia; ^6^ MBioMe - Mine Site Biomonitoring using eDNA Research Group, Trace and Environmental DNA (TrEnD) Laboratory, School of Molecular and Life Sciences, Curtin University, Bentley, Western Australia 6102, Australia; ^7^ School of Molecular and Life Sciences, Curtin University, Bentley, Western Australia 6102, Australia; ^8^ Centre for Crop and Disease Management, School of Molecular and Life Sciences, Curtin University, Bentley, Western Australia 6102, Australia; ^9^ Environmental Science and Research (ESR), Porirua 5022, New Zealand

**Keywords:** invertebrate-derived DNA, metabarcoding, terrestrial vertebrate, biomonitoring

## Abstract

Invertebrate-derived DNA (iDNA) metabarcoding from carrion flies is a powerful, non-invasive tool that has value for assessing vertebrate diversity. However, unknowns exist around the factors that influence vertebrate detections, such as spatial limits to iDNA signals or if detections are influenced by taxonomic class or estimated biomass of the vertebrates of interest. Using a bulk-collection method, we captured flies from within a zoo and along transects extending 4 km away from this location. From 920 flies, we detected 28 vertebrate species. Of the 28 detected species, we identified 9 species kept at the zoo, 8 mammals and 1 bird, but no reptiles. iDNA detections were highly geographically localized, and only a few zoo animals were detected outside the zoo setting. However, due to the low number of detections in our dataset, we found no influence of the taxonomic group or the estimated biomass of animals on their detectability. Our data suggest that iDNA detections from bulk-collected carrion flies, at least in urban settings in Australia, are predominantly determined by geographic proximity to the sampling location. This study presents an important step in understanding how iDNA techniques can be used in biodiversity monitoring.

## 1. Introduction

Monitoring species diversity and abundance is crucial to managing and informing efforts to prevent biodiversity loss [1]. Information obtained from monitoring can improve understanding of population trajectories and extinction risk, the response of species to threatening processes and evaluate management effectiveness [1–3]. Most current monitoring methods for terrestrial vertebrates rely on directly observing species in an area or the traces they may leave behind, such as hair, footprints or scat [4]. This can be time- and resource-intensive and require the mobilization of experts over long periods [5]. Several methods, such as camera traps and passive acoustic loggers, have been developed to reduce the direct human costs associated with monitoring ecosystems. Recently, DNA metabarcoding of environmental substrates, such as soil [6,7], water [8–10], flowers [11] and air [12,13], has been used to monitor vertebrate biodiversity. An extension of this is the use of invertebrate-derived DNA (iDNA), whereby vertebrate diversity is monitored using invertebrates that feed on the blood, flesh or scat of vertebrates and therefore carry their DNA signal [14–17].

Species detection through iDNA metabarcoding works by extracting DNA from invertebrate samples, amplifying the DNA using nucleotide-labelled primers that target vertebrate sequences, sequencing the DNA on high-throughput platforms and identifying the resulting sequencing against reference sequence databases [15,18]. This method is an efficient and cost-effective way to monitor vertebrate diversity in remote or difficult terrain [19]. Unlike camera traps, which are biased towards detecting larger-bodied terrestrial mammals as their size can trigger the cameras more easily [20], iDNA can be used to detect smaller-bodied taxa, including arboreal or non-mammal species [21]. Studies using iDNA have focused on invertebrate groups such as leeches [17,22], sandflies and mosquitoes [19,23], dung beetles [24] and carrion flies [15,19,21,25] as ‘samplers’. Compared with mosquitoes and sandflies, carrion flies (families Calliphoridae and Sarcophagidae) have been found to be the most effective iDNA sampler at capturing vertebrate species richness [19]. Unlike leeches, which can retain vertebrate DNA in their guts for months at a time [26], the short-lived persistence of DNA transported in carrion flies (24−96 h) [27] makes them ideal candidates for measuring current vertebrate diversity and distributions. Furthermore, carrion flies are widespread in many different environments and are easy to sample [28]. Studies using carrion flies for vertebrate detection have so far been undertaken on mammal communities in Africa [15,29], Australia [30], South America [31] and Asia [25,32] and vertebrate communities in urbanized areas in Europe [33] and North America [34].

Despite the utility of carrion fly iDNA for vertebrate monitoring, some aspects of its use are yet to be explored. Exploring biases in detectability is an important part of calibrating this technique for use in biodiversity monitoring, and here we examine three factors: distance, taxonomic group and biomass, which all could have an influence on iDNA detection. Firstly, carrion flies are very mobile, with some species of calliphorid flies documented dispersing up to 63.5 km from a source [35]. However, a recent study suggests that mammalian iDNA signals are localized around sources of mammalian biodiversity [30]. Furthermore, carrion flies are opportunistic feeders, and, unlike haematophagous invertebrates or mosquitoes, have yet to be shown to have species preference [15,19]. It is known that some classes of vertebrates, such as reptiles, can have poor DNA shedding rates and lower detection in eDNA studies [36]. Similarly, different animal classes have been found to have different shedding rates in aquatic environments [37]. However, it is unknown how this would impact detections from iDNA in terrestrial environments and whether shedding rate or fly preference towards different groups of animals would have an influence on detection. Moreover, the eDNA signal in samples taken from aquatic environments can be influenced by the biomass of animals in the area [38]. In terrestrial environments, it was more probable to detect larger vertebrates and those captured multiple times in the camera traps using sediment samples [7]. It is unknown if, in the same way, the biomass of animals in an area can influence detection via iDNA, that is, if the mean mass of individuals of a particular species is greater than that of others, whether that would provide more readily available food sources for flies and increase detection rates.

In this study, we used iDNA metabarcoding from carrion flies to detect vertebrate species and examined several factors that could impact their detectability using a bulk-collection method and a non-destructive extraction procedure for collecting and processing carrion flies as in Fernandes *et al.* [30]. While this methodology has been used before to detect mammals and birds from insects [16,30], it is unknown how factors such as distance, biomass of animals within a given area or taxonomic class would influence iDNA detections. We used the controlled environment of a zoo with a known vertebrate species diversity, abundance and location. Firstly, we aimed to determine the approximate geographic range of an iDNA signal. We predicted that the detections of zoo taxa would decline with distance from their enclosures. We then set out to assess the influence of taxon and estimated biomass on the detectability of vertebrates through carrion fly iDNA. From previous iDNA studies, mammals and birds are detected more frequently than reptiles, so we expected these trends to be reflected in our data [19,25]. We also predicted that animal populations representing higher estimated biomass would be detected more frequently and at farther distances than animals from populations with lower estimated biomass based on the increased availability of faeces as a food source for carrion flies.

## 2. Methods

### 2.1. Study site

**Figure 1.**
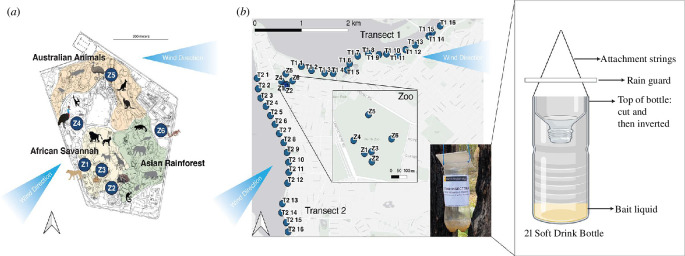
(*a*) Map of Perth Zoo showing the locations of carrion fly traps within the zoo (Z1–Z6). Major ‘animal zones’ designated by Perth Zoo that correspond to the groups of animals kept within the zoo are illustrated in colour with corresponding labels: African Savannah, Asian Rainforest and Australian Animals. Animal enclosures where the fly traps were put into are included in colour, other zoo animals detected in this study are indicated as black silhouettes, and zoo animals located in the same animal zone but not detected are shown as grey silhouettes. Prevailing wind directions are marked as blue arrows. (*b*) Map showing the sampling locations (dark blue circles) spaced approximately 250 m apart along 4 km transects away from Perth Zoo (16 sampling locations per transect). The inset map shows the locations of the fly traps within the zoo. Prevailing wind directions are marked as blue arrows. Inset image shows a carrion fly sampler with pop-out diagram of the trap construction. Diagram created with Biorender.com.

This study was conducted within the Perth metropolitan region in southwest Western Australia, using baited plastic bottle traps to capture flies (figure 1*b*, inset) during the spring/summer season (November–December 2020). The sampling areas were distributed along the Swan River/Derbarl Yerrigan (figure 1*b*). This area is predominantly residential, interspersed with medium- and high-density housing, commercial buildings and parklands. To measure the distance of iDNA detections from a source point, the Perth Zoo was chosen as the source of a known diversity and abundance of exotic animals not native to the local area.

Perth Zoo is a 17 ha zoological park located in the Perth metropolitan area; it houses 164 animal species. The zoo has created ‘animal zones’ that group the displayed animals according to their geographical region of origin. There are three main open-air animal zones: the ‘African Savannah’ that houses animals found in the African continent or surrounding islands; ‘Australian Animals’ that houses native Australian animals from Australia; and the ‘Asian Rainforest’ that houses animals found in the Asian continent (figure 1*a*). While some species are kept in indoor enclosures, 98 are displayed with access to an outside enclosure. These animals can thus more readily interact with flies. They comprise 3 amphibian, 41 bird, 40 mammal and 14 reptile taxa (electronic supplementary material, table S1).

### 2.2. Sample collection

A bulk-collection method was employed to capture flies. Fly traps were made from 2 l plastic bottles which had the top and nozzle cut off and then inverted into the bottle (figure 1*b*). Before initial deployment, traps were sterilized for 30 min with a 20% bleach solution. Gloves were used to handle all traps. Flies could easily enter the trap but could not fly vertically upwards to leave. Approximately 20–30 ml of a commercial, organic, fly bait (‘Magna Fly Bait’, GEPRO, Navel Base, Australia) was put into each trap.

Six trapping locations were identified inside the zoo, near open-air enclosures, which were accessible to flies, these locations were near the radiated tortoise (*Astrochelys radiata*), Galapagos tortoise (*Chelonoidis nigra*), emu (*Dromaius novaehollandiae*), southern cassowary (*Casuarius casuarius*), numbat (*Myrmecobius fasciatus*) and lion (*Panthera leo*) enclosures and traps were placed less than 5 m away from animal enclosures. At each of the six trapping locations in the zoo, three traps were deployed.

Outside the zoo, fly traps were set up along two transects away from the zoo. Fly traps were set every 250 m up to 4 km away from the zoo along highways, roads and residential parkland (figure 1*b*). Here, 4 km is assumed to be the average daily flight distance of most calliphorid fly species [28,35,39]. The transects followed the coast of the peninsula of south Perth in two directions to account for prevailing wind directions, which can be a key factor for carrion fly dispersal [40]. During the sampling period, Perth experienced a strong easterly during the day with a southwesterly sea breeze in the afternoons.

Traps were checked both within and outside the zoo and flies were collected every 72 h over 21 days. This yielded seven possible sampling time points for each of the 18 deployed traps in the zoo and 32 deployed traps outside the zoo. Following each collection, traps were thoroughly rinsed with bottled water, but not sterilized before the next sampling. The bait was replaced, and then the same fly trap was used at each sampling point. All flies collected from a single trap at a single time point were considered one sample and extracted together. As not every sample contained flies at every collection point, in total, we collected 123 fly samples across the sampling period. Samples were transported chilled on ice and then frozen at −18˚C upon arrival at the laboratory.

### 2.3. Sample processing and DNA extraction

Each sample contained an average of 12 flies (s.e. ± 2 flies). The minimum number of flies in a sample was 1 and the maximum was 54 (electronic supplementary material, table S2). Samples were thawed, and flies were gently washed with ultrapure water to remove any traces of fly bait before being stored in 70% ethanol at −18˚C, prior to DNA extraction. Before DNA extraction, ethanol was poured off the samples and samples were gently rinsed with ultra-pure water. DNA was extracted using a non-destructive DNA digestion protocol [41] that lyses and releases cellular DNA without damaging the exoskeleton of the flies. The digestion buffer (as in [41]), was added to the cleaned flies with enough volume to cover them (approx. 1–3 ml) and these were digested overnight at 56˚C. This was followed by purification and concentration using a DNEasy Blood and Tissue kit following the manufacturer’s instructions (Qiagen, Venlo, The Netherlands) modified for 400 µl of digestion fluid and a 200 µl elution volume in EB buffer. Negative extraction controls were included for every 23 samples. DNA extracts were stored at −18˚C in ‘Lo-bind’ Eppendorf tubes.

### 2.4. PCR amplification and sequencing

Two metabarcoding primer sets were used to target two taxonomically informative markers. These were chosen as relatively short markers were needed to ensure PCR amplification despite potential DNA degradation due to digestion within the fly digestive tract. A vertebrate-specific primer set was selected (vertebrate 12S), which targets the mitochondrial 12S rRNA region (F: 5′-TAGAACAGGCTCCTCTAG-3′; R: 5′-TTAGATACCCCACTATGC-3′; 98 bp [42]) and a mammal-specific primer set was selected (mammal 16S), which targets the mitochondrial 16S rRNA region (F: 5′-CGGTTGGGGTGACCTCGGA-3′; R: 5′-GCTGTTATCCCTAGGGTAACT-3′; 130 bp [43]).

Prior to metabarcoding, quantitative PCR (qPCR) was carried out on all 123 sample extracts to assess the amplification efficiency and the presence of PCR inhibitors in the samples using a StepOnePlus Real-Time PCR System (Applied Biosystems, Waltham, MA, USA). This was done on a dilution series for each sample extract (undiluted, 1:2 and 1:5). qPCRs were carried out in 12.5 µl reactions containing: 1 U of AmpliTaq gold, 1× PCR Gold Buffer and 2 mM MgCl_2_ (all from Applied Biosystems), 0.4 mg ml^−1^ bovine serum albumin (Fisher Biotec, Wembley, Australia), 0.25 mM dNTPs (Astral Scientific, Taren Point, Australia), 0.4 µM of each forward and reverse primer, 0.6 µl of 1/1000 SYBR Green (Invitrogen, Waltham, MA, USA) and 1 µl of template DNA. The qPCR programme for the vertebrate 12S assay was as follows: 95˚C for 5 min, followed by 45 cycles of denaturation at 95˚C for 30 s, 60˚C for 30 s and 72˚C for 45 s, with a final elongation at 72˚C for 10 min. For the mammal 16S assay, the qPCR programme was as follows: 95˚C for 5 min, followed by 45 cycles of 95˚C for 30 s, 57˚C for 30 s and 72˚C for 45 s, with a final elongation at 72˚C for 10 min. Negative extraction controls, qPCR, ‘fly bait’ and positive (quenda; *Isoodon fusciventer*) controls were included alongside the sample extracts during DNA quantitation. Quenda was used as a positive control as DNA from the species is known to amplify well using our laboratory workflows. Positive control samples used in the qPCR screening were not sequenced. This was because quenda is a wild species found in the local area and any contamination from a positive control would be hard to tease apart from detections of the mammal if it was found in the samples.

Following qPCR screening, metabarcoding was carried out. For each sample, sample extract dilutions that had shown the highest level of amplification and minimal inhibition were amplified using qPCR with ‘fusion primers’, called a one-step approach [44]. The fusion primers consisted of the metabarcoding primers, labelled on the 5′ end of both the forward and reverse primers with 6–8 nucleotide long molecular identification (MID) tags followed by Illumina sequencing adaptors. Amplifications were carried out so that each sample was tagged with a unique combination of forward and reverse MID tags. All MID tag combinations were not previously used in the laboratory. To minimize the risk of contamination, qPCR reactions were prepared in an ultra-clean pre-PCR laboratory free from extracted or amplified DNA and DNA was added in another DNA extraction laboratory free from amplicons.

All sample extracts, extraction and qPCR negative controls, and a ‘fly bait’ control were amplified in duplicate using the qPCR conditions mentioned above. Duplicates carried the same MID tags. PCR products were pooled in approximate equimolar concentrations into 20 sample pools informed by the qPCR amplification curves. Negative controls were spiked into libraries, and the ‘bait’ control was blended in equimolarly with other samples. DNA in the pools was then quantified using a QIAxcel Advanced System (Qiagen) with a QIAxcel DNA High-Resolution Kit. Sample pools were then pooled into one sequencing library in equimolar ratios. The library was size-selected using a Pippin Prep 2% agarose Marker B cassette (Sage Science, Beverly, MA, USA) for 150–600 bp fragments to eliminate primer dimer. The library pool was then purified using a QIAquick PCR purification kit (Qiagen) as per the manufacturer’s instructions with the addition of a 5 min incubation at room temperature before elution. The purified library was eluted in 40 µl and quantified with a QuBit (Invitrogen) using double-stranded DNA high-sensitivity reagents. The library was sequenced on an Illumina MiSeq (Illumina, San Diego, CA, USA) flowcell using a single-end 300-cycle V2 kit as per the manufacturer’s directions.

### 2.5. Bioinformatics and sequence processing

Sequence data for each primer set were processed separately. Sequences were demultiplexed using ‘obitools’ [45], with no mismatches in the MID/primer sequence allowed. Sequences were then length filtered for a minimum length of 50 bp. The ‘DADA2’ package [46] in R v. 3.6.3 [47] was then used to further quality filter sequences. Sequences were quality filtered with a max expected error of 2, and those identified as chimaeras were removed. Sequences were then dereplicated to produce amplicon sequence variants (ASVs). ASVs were matched to the NCBI GenBank reference database (www.ncbi.nlm.nih.gov/genbank/) using the Basic Local Alignment Search Tool (BLAST) for taxonomic assignment using a high-performance cluster computer (Pawsey Computing Centre, Perth, Western Australia, Australia). Taxonomic assignments were made to the lowest common ancestor (LCA) using the LCA script from eDNAFlow [48] with a minimum query coverage of 100% and identity threshold of 95%. Where the absolute value of the difference between percentage identity of ASVs was <1, species taxonomy was not returned, and the ASV was assigned to the closed common ancestor. The following ‘contaminant’ ASVs were filtered from the dataset using the ‘phyloseq’ package [49]: from the vertebrate 12S assay, within the fly bait control, 178 ASVs (3 358 815 sequences) were identified as cow (*Bos taurus*), pig (*Sus scrofa*) and sheep (*Ovis aries*), and these ASVs were removed from further analyses. Furthermore, 10 ASVs (2132 sequences) were removed from the dataset as they were found in the negative controls; these ASVs were identified as belonging to cow and chicken (*Gallus gallus*), both common laboratory contaminants. Additionally, 6 ASVs (27 196 sequences) in the 12S dataset that were identified as human (*Homo sapiens*) were removed.

For the mammal 16S assay, within the fly bait control, 195 ASVs were identified as cow, pig and sheep, and these specific ASVs were removed (4 693 199 sequences). In addition, 9 ASVs (143 260 sequences) identified as cow and chicken were found in the negative controls and were removed from the dataset. Finally, 7 ASVs (18 596 sequences) in the 16S dataset were identified as human and were removed. The ASVs were then agglomerated at a species level, retaining ASVs identified at a higher taxonomic level. For analysis requiring ASV tables from both assays to be combined, ASV tables were merged whereby if the vertebrate species identity was identical between both assays the ASVs were agglomerated. The ASV tables from each primer set were kept separate for other analyses.

### 2.6. Statistical analysis

All statistical tests were run on R v. 3.6.3 [47]. All maps were created using QGIS v. 3.2.3-‘Bonn’ [50]. To investigate the effectiveness of our sampling effort, the ASVs from both assays were combined. Species accumulation was estimated using extrapolation and rarefaction curves based on the Chao1 estimator, and configured at 40 knots and 300 bootstraps to determine confidence intervals using the ‘iNEXT’ package [51] and ‘iNEXT Online’ [52,53]. For species accumulation, we used repeated samplings of individual traps to account for the re-use of traps between each sampling (*n* = 31 for all sites and *n* = 6 for within the zoo). We only counted species from the zoo or ‘wild’ in our estimates. From the species that were detected at the zoo and along the transects (*n* = 4), the number of sequences found at each distance (m) was assessed using a negative binomial generalized linear model using the package ‘MASS’ [54]. Sequences are not compared between species, but rather together from sequences of zoo animals located in the zoo (0 m) to 4 km away.

We then assessed the influence of taxonomic group and biomass on the detectability of zoo taxa. First, for each species present at the zoo, the estimated total biomass of species at the zoo was calculated based on the number of individuals of the species kept at the zoo multiplied by the average mass of an individual of that species based on a literature search (electronic supplementary material, table S1). Then, two logistic regression models with binomial distribution were used to test the probability of detection of a species kept at the zoo from carrion fly iDNA (factor of presence/absence of the species in the metabarcoding dataset), the first with the number of species in each taxon group (factor with four levels: amphibian, bird, mammal and reptile) was created using the species detections at the zoo from the general vertebrate 12S primer set [42], to avoid any confounding effects of a mammal-specific primer set increasing the numbers of mammals detected. The second model was created with the total biomass of each species as a continuous explanatory variable, and the same factor of presence/absence. This was conducted on the combined species detections from both assays (vertebrate 12S and mammal 16S).

## 3. Results

In total, 920 flies were collected and pooled into 123 samples. Over 13 000 000 reads were generated from these flies, with an average of 52 546 (±4003 s.e.) per sample from the mammal 16S assay and 61 254 (±7265 s.e.) per sample from the vertebrate 12S assay. The use of passive traps, where flies can interact with fly bait, meant that the bait signal (cow, pig and sheep) was high across samples. After removing ASVs associated with bait, there was a significant reduction in the number of sequences, with an average of 7.5% of sequences retained overall from samples across both primer sets. Within these, 50 ASVs not associated with the fly bait were detected. These included vertebrates across four taxonomic classes spanning species kept in the zoo, wild animals, domestic animals and ‘other’ (table 1). For animals classed as ‘other’, these are taxa of unknown origin. They could be species found in human refuse or used as feed for zoo animals. As it is not possible to determine the true origin of these taxa because the content of zoo animal feed or refuse is unknown, they have been classified as ‘other’. Combining detections from the two assays resulted in 87 samples with vertebrate detections out of the 123 samples analysed; 64 out of 90 samples along the transects and 23 out of 35 samples from the zoo. On average, there were 5 (±0.4 s.e.) vertebrate ASVs per sample. There were 19 samples in total that contained zoo taxa, 8 samples from the zoo and 11 samples along the transects.

**Table 1.** The vertebrate species detected through metabarcoding of carrion fly iDNA, not including humans or animals found in bait or as laboratory contamination, and the number of detections across samples (in parentheses). At the zoo, this number is from six total samples. At Transect 1 and Transect 2, this is from a maximum of 16 samples from each transect. Common names are included where appropriate. The detection location of each animal is noted as follows: Zoo (Z), Transect 1 (T1) and Transect 2 (T2). Further notation is included for animals not housed or inhabiting the zoo. This notation is for wild animals (*), domestic pets (†), or an animal whose origin could not be determined, noted as other (‡).

class	order	family	genus	species	common name	location and detections
zoo animals						
Aves	Pelecaniformes	Ardeidae	*Ardea*	*modesta*	eastern great egret	Z(3)
Mammalia	Carnivora	Canidae	*Lycaon*	*pictus*	African painted dog	Z(3), T2(4)
Mammalia	Carnivora	Felidae	*Panthera*	*leo*	lion	Z(4), T1(5), T2(5)
Mammalia	Carnivora	Viverridae	*Arctictis*	*binturong*	binturong	Z(1)
Mammalia	Diprotodontia	Macropodidae	*Macropus*	*fuliginosus*	western grey kangaroo	T2(1)
Mammalia	Diprotodontia	Macropodidae	*Osphranter*	*rufus*	red kangaroo	Z(1)
Mammalia	Primates	Cebidae	*Saguinus*	*oedipus*	cotton-top tamarin	Z(1), T2(1)
Mammalia	Primates	Cebidae	*Saguinus*			Z(1)
Mammalia	Primates	Cercopithecidae	*Papio*	*hamadryas*	hamadryas baboon	Z(2)
Mammalia	Primates	Hylobatidae	*Hylobates*	*moloch*	Javan gibbon	Z(1), T1(2), T2(1)

**w**ild*, domestic^†^ or other^‡^ animals						
Actinopteri	Perciformes	Centropomidae	*Lates*	*calcarifer*	barramundi^‡^	T2(1)
Actinopteri	Scombriformes	Scombridae^‡^				Z(2), T1(2), T2(4)
Amphibia	Anura	Hylidae	*Litoria*	*moorei*	motorbike frog*	Z(2)
Amphibia	Anura	Myobatrachidae	*Pseudophryne*	*guentheri*	Gunther’s toadlet*	Z(1)
Aves	Pelecaniformes	Ardeidae	*Nycticorax*	*caledonicus*	Nankeen night heron*	Z(1)
Epidosauria	Squamata	Scincidae	Hemiergis*			T1(1)
Mammalia	Artiodactyla	Bovidae	*Capra*	*hircus*	goat^‡^	Z(1), T1(1), T2(1)
Mammalia	Artiodactyla	Cervidae	*Cervus*		deer^‡^	T1(1), T2(1)
Mammalia	Carnivora	Canidae	*Canis*	*lupus familiaris*	domestic dog^†^	Z(5), T1(9), T2(7)
Mammalia	Carnivora	Felidae	*Felis*	*catus*	domestic cat^†^	Z(1), T1(1), T2(1)
Mammalia	Diprotodontia	Phalangeridae	*Trichosurus*	*vulpecula*	brush-tailed possum*	Z(4), T1(1), T2(4)

**Figure 2.**
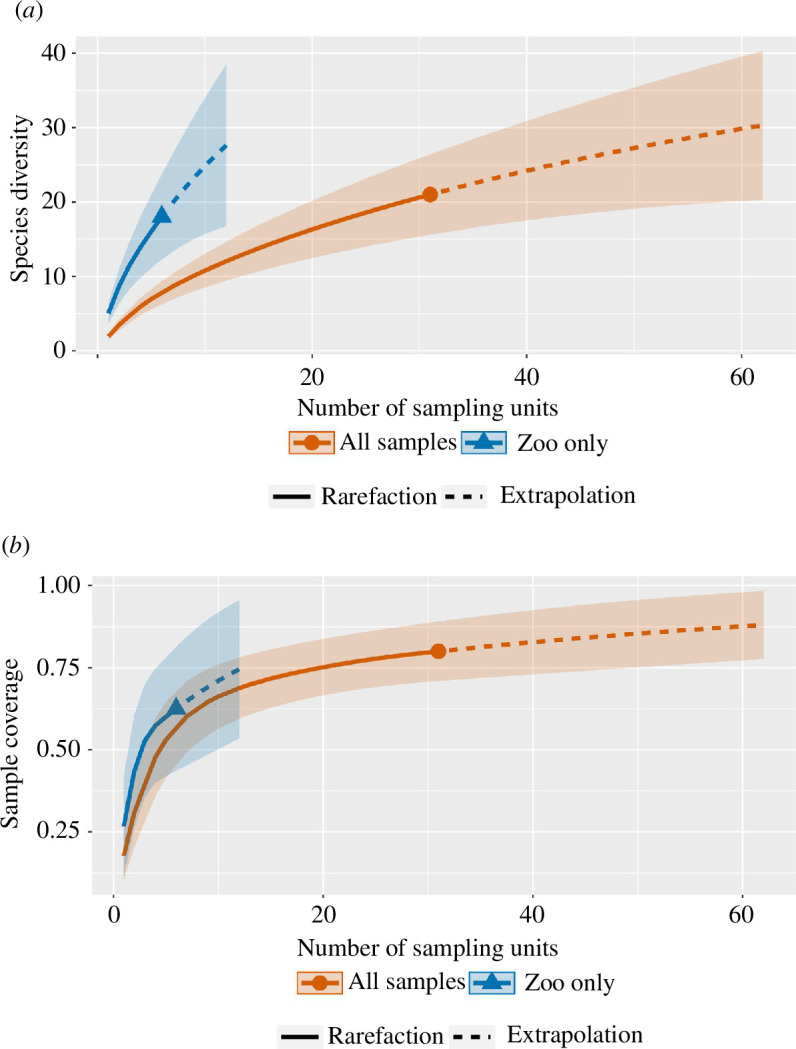
Species accumulation curves for zoo and wild animals detected from carrion flies caught at samples within the zoo (blue triangle), and all samples collected from all samples (orange circle). (*a*) Sample size-based rarefaction and extrapolation sampling curve. (*b*) Sample completeness curve.

The obtained sample size-based rarefaction and extrapolation curves (figure 2*a*) and sample completeness species accumulation curves (species accumulation) (figure 2*b*) were indicative of inadequate sampling effort (electronic supplementary material, tables S3 and S4). The insufficient coverage of the target ecosystem led to an underrepresentation of species diversity, especially within the zoo samples, resulting in incomplete and potentially biased curves (electronic supplementary material, tables S3 and S4).

**Figure 3.**
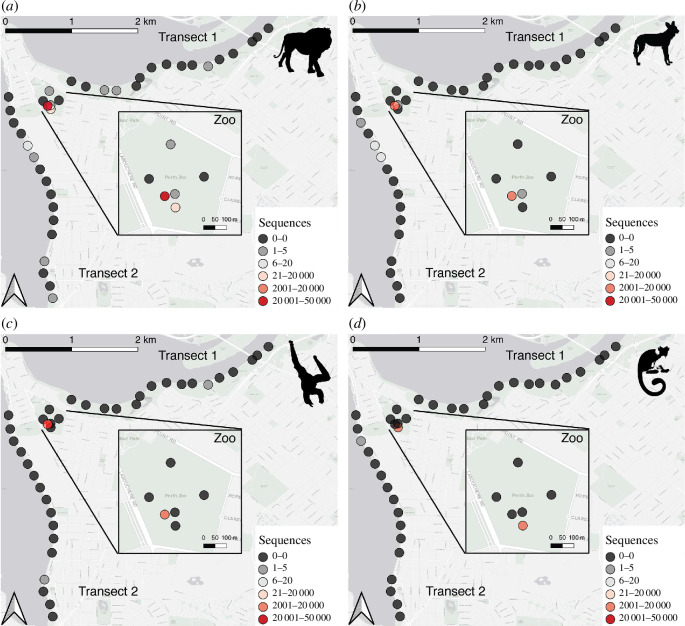
Number of sequences of the four zoo-resident mammal species detected both in the zoo (inset) and along the transects: (*a*) *Panthera leo* (lion), (*b*) *Lycaon pictus* (African painted dog), (*c*) *Hylobates moloch* (Javan gibbon) and (*d*) *Saguinus oedipus* (cotton-top tamarin). The colour of the circles indicates the total number of sequences detected from samples at the transect point across all sampling days. Silhouettes of the detected animals are included.

Within the 64 bulk carrion fly samples collected along the two 4 km transects, only four of the taxa present in the zoo were detected along the transects, these were all mammal species: lion, African painted dog (*Lycaon pictus*), Javan gibbon (*Hylobates moloch*) and cotton-top tamarin (*Saguinus oedipus*) (figure 3). One species, the western grey kangaroo (*Macropus fuliginosus*), was found only along Transect 2, approximately 1.5 km from the zoo, and was detected from only two sequences. This species was not detected within the zoo, despite being kept there. Along the transects, three species (lion, African painted dog and cotton-top tamarin) were detected from both assays. The Javan gibbon was only detected using the mammal 16S assay, and the western grey kangaroo was only detected using the vertebrate 12S assay. The number of sequencing reads from these species decreased as samples were collected at greater distances from the zoo. For example, within the samples from within the zoo, a total of 8889 sequences of African painted dog were detected, while only 16 sequences were detected in samples collected 1.25 km from the zoo along Transect 2 (figure 3*b*). The furthest detection of a zoo animal was in a sample collected 4 km from the zoo in Transect 2 where a single sequence of lion was detected (figure 3*a*); however, under more stringent filtering conditions this detection would not be considered likely a true positive detection. We conducted a negative binomial regression to test the relationship between distance from the DNA source and sequence number, and found a significant negative relationship (Model *χ* ^2^(1) = 125 102, *p* < 0.0001), with a 98.9% reduction in sequence number from the source (CI = −12.73 to −1.63). However, because we lack replication across all data points and across multiple species from the zoo, this result must be taken with some caution.

From the vertebrate 12S assay only, five species kept at the zoo were detected: one bird species (*Ardea modesta*) of the 40 bird species with access to outdoor enclosures and four mammal species (lion, cotton-top tamarin, African painted dog and western grey kangaroo) of the 40 mammal species with access to outdoor enclosures. With the addition of the mammal 16S assay, a further four mammal species found within the zoo could be detected: Javan gibbon, binturong (*Arctictis binturong*), red kangaroo (*Osphranter rufus*) and hamadryas baboon (*Papio hamadryas*). Using the data from the vertebrate 12S assay only, to avoid the confounding effects of a mammal-specific assay, the likelihood of being detected did not differ between taxon (deviance explained = 0.0466; d.f. = 4,94; *p* = 0.742). From the 98 species at the zoo, mammals made up 87.6% of the total biomass, followed by reptiles at 9.7% and birds at 2.7%. Some of the mammals contributing to the high total biomass such as the white rhinoceros (*Ceratotherium simum*) (~4000 kg) or giraffe (*Giraffa camelopardalis*) (~4125 kg) were not detected, and neither were mammals with very small total biomass, such as the pygmy marmoset (*Cebuella pygmaea*) (~3.5 kg) (electronic supplementary material, table S2). The total biomass of the detected zoo animals ranged from ~4.3 kg (cotton-top tamarin) to ~440 kg (African hunting dog). There was no relationship between biomass and the likelihood of being detected (deviance explained = 0.0007; d.f. = 1,97, *p* = 0.864).

## 4. Discussion

The iDNA is increasingly used to assess vertebrate species diversity [19,29–31,55]; therefore, it is vital to examine the factors that influence the detection of species using this technique. Here, we used the same bulk-collection method as in Fernandes *et al.* [30] to collect and process flies, with two metabarcoding assays to detect vertebrate taxa from carrion flies collected from within the Perth Zoo and two transects extending away from the zoo. Despite the presumed mobility of carrion flies, we found that the signals from iDNA detections of zoo animals were predominantly geographically localized to the zoo, with much fewer sequences from zoo animals detected outside the zoo. We could not detect all species of zoo animals with our iDNA techniques, with no discernible pattern of non-detection related to biomass or taxonomic class. Further studies are needed to understand whether this limited detection is due to the target animals, the physical setting of the zoo, the sampling strategy, or another untested variable.

### 4.1. Distance from a source of DNA

Detections of zoo vertebrate species from carrion fly iDNA were highly localized, even though previous studies have shown that carrion flies can travel 2.2–6.4 km over a 24 h period, depending on the species [28,35,56]. However, these studies aimed to discover the maximum dispersal distance from a release event, not the distance a fly travels from a food source. In the presence of a food source, it is possible to distinguish carrion fly communities within 300 m [57]. Further, these studies have not shown how structural components of the environment may affect movement (e.g. in an urban setting, the impacts of roads or buildings). Within the samples collected from the zoo, zoo animal species were detected with relatively high numbers of sequences, indicating a highly localized signal that weakens with distance from the DNA source. This supports previous studies that indicate the localization of mammalian iDNA signals around sources of mammalian biodiversity [30]. A recent leech iDNA study also found that detection probability improved closer to the vertebrate DNA source [58].

It should be noted that the number of sequences cannot be interpreted without the caveat that PCR and sequencing conditions can skew sequence abundance [59,60], especially in samples of mixed taxa. Thus, we did not compare sequence numbers between species. However, from the overall data, there is an observed pattern of decline in the number of sequences from zoo taxa further away from the zoo. While zoo animals could still be detected up to 4 km away from the zoo, these were represented by only very few sequences (<20). More stringent filtering of ASVs would remove these detections completely from the dataset. Therefore, this interpretation of detection distance reflects a much narrower scale of localization from carrion fly detections. However, there is variability in the dispersal ability of different fly species [61], and targeting some species of flies over others may change the spatial resolution of iDNA detections. Further investigation using this technique, including examining the effect of habitat type, fly species, climatic and seasonal conditions, which can all impact fly dispersal patterns [28,39,61], is required to test the broader applicability of our findings.

### 4.2. Detection of different animal taxa

We found no significant relationship between the taxonomic group of animal and detectability, but this may be due to generally poor detection success for all taxa in the zoo. Our incomplete sampling curves highlight the need for a more comprehensive sampling strategy to effectively capture the true richness of the species present within an area, especially in areas with high species diversity and abundance (like a zoo setting). However, even discounting the results of the mammal-specific assay, more mammal species were detected than other groups in our limited dataset. Even when traps were located in enclosures close to reptile and bird species, detection success was poor. These results are comparable to those of other studies using different eDNA substrates to detect vertebrates, such as air [13], water [8,62] and soil [7], where mammals were most commonly represented in detections, and reptile species were poorly represented or completely absent. The difficulties associated with detecting reptiles through metabarcoding could possibly be explained by the relatively lower deposition rate of reptile DNA compared to other classes of vertebrates [36,63]; however, tortoise faeces, which would theoretically provide a food source for carrion flies, was observed in the enclosures of both species.

The detection of birds from carrion fly iDNA from our study was poor, with only two bird taxa detected. Given that the detections from carrion flies are predominantly related to feeding on faeces in the zoo [34], the limited detections from the birds and the reptiles in the zoo samples could be related to the presence of free DNases in avian faeces [64] or faecal microbes in reptile faeces [65], both of which are known to rapidly degrade DNA. However, the absence of wild bird species at non-zoo locations, where flies would also theoretically interact with carrion, cannot solely be explained by these factors. While it is possible to obtain DNA from reptile faeces to conduct monitoring and analysis [65–67], this is usually dependent on the collection of fresh samples [65]. Additionally, iDNA degrades quickly within the digestive tract of invertebrates [27,68]. It is worth noting that despite attempts to detect various different classes of animals, overall detection was poor throughout the study, and no significant differences in detection were observed between taxonomic classes. Additionally, while other studies using carrion fly iDNA have reported successful detection of bird species using the same primers [19,30], our findings did not align with these results. The discrepancy in our findings could potentially be attributed to variations in sampling techniques, environmental factors or species composition across different study locations.

In this study, we used the same metabarcoding primer sets as used in previous carrion fly iDNA studies [15,23,29,33], which included a general vertebrate primer [42] and a mammal-specific primer [43]. While the general vertebrate primer set [42] can detect mammals, birds, and amphibians and, *in silico*, matched against reptiles and birds, they may exhibit preferential binding towards mammals in a mix of DNA and may have preferentially bound to the fly bait DNA. While we detected more mammals with a mammal-specific primer set, we could only detect a fraction of the total mammal biodiversity at the zoo. Therefore, while including a more specialized taxon-specific reptile or bird primer set (see [11]) may increase the biodiversity detected from these taxon groups, our results show that our sampling methodology would need to be improved to increase detections overall. This could be accomplished by increasing sampling intensity through a longer sampling regime or by installing more traps. Additionally, some methodological improvements, such as increasing the sequencing depth of samples [69], using an inorganic bait solution or a trap that separates the bait from the collection chamber (as in [19]) or adjusting the extraction procedure to release more non-fly DNA through maceration of fly samples (as seen in previous studies, for example [29]), may have improved the identification of zoo vertebrate species.

We found that the DNA associated with fly bait overwhelmed the vertebrate signals, and we retained only 7.5% of sequences as non-bait taxa. Our findings were similar to those of Calvignac‐Spencer *et al.* [15], who used a mammalian bait (but did not use a collection fluid) and retained only 12% of their sequences. Furthermore, attempts to wash the bait off the flies could have resulted in the loss of iDNA signals on the outside of the flies (i.e. on the legs or body). To overcome this issue, some iDNA studies have used blocking primers, enabling the detection of more ‘target’ species to reveal more ‘wild’ diversity by minimizing the detection of domestic animal signals or the animal used as a fly bait [29,33]. Blocking primers work by selectively blocking the DNA of a species whose amplification can potentially mask the presence of DNA from other species, for example, blocking the amplification of human DNA [70]. Blocking primers are common in dietary studies when the amplification of the host DNA can overwhelm the signal from the dietary prey items [71] or avoid human contaminant sequences [6]. In this study, we had no prior knowledge of the bait contents, which limited the use of blocking primers. Furthermore, blocking primers can also block more taxa than just their intended target, thereby introducing bias in survey results [72]. Therefore, improving trap design to avoid the interaction between flies and the bait/creating a barrier between the bait and collection fluid may be more beneficial than blocking primers to reduce the signal. Trap designs may follow options such as Massey *et al.* [19] that use a barrier between the bait and then cold to ‘stun’ insects for removal from the traps so they can be put into preservation/collection fluid.

### 4.3. Impact of biomass on detection

In contrast to the findings of studies using water or air as the sampler of vertebrate diversity [13,73], we found no correlation between the detections of our species and increasing biomass. However, increasing the sample size or sequencing depth may reveal different findings in future studies. Our results do not support the idea that larger animals are more likely to be detected than smaller ones, which is related to detection success from sediment sampling [7]. This is important as many smaller-bodied, arboreal or volant mammals, such as rodents or bats, are difficult to detect using approaches such as camera trapping [21]. A possible explanation for this finding is that flies do not move far from a food source, so a fly’s proximity to a sampling point would be the main driving factor of iDNA detection rather than an animal’s total biomass. Physical barriers may also impede movement or odour cues that would attract carrion flies to a potential food source [74]. In our study, many of the mammals with the largest total biomass, such as elephants (*Elephas maximus*), giraffes (*G. camelopardalis*) and zebras (*Equus quagga*), which were all undetected, were located in sunken enclosures that might physically restrict the movement of flies from this area to the fly traps in other locations. It is also possible that the faeces of large herbivorous animals are less attractive to the flies than the faeces of carnivores or omnivores which may have different nutritional value or moisture content. For example, certain species of dung beetles have been found to exhibit differential preferences towards different animals’ faeces [75]. In the future, considering the taxonomical diversity of flies in the trap would be a valuable addition, increasing the interpretability of the data collected. As we used a non-destructive technique to sample flies [41], with the correct expertise, this would be possible using our methodologies.

### 4.4. Methodological considerations

The current study represents an important step in exploring the biases inherent in carrion fly iDNA studies. However, it is imperative to acknowledge some caveats in the methodologies. We present a carrion fly field sampling technique that does not require significant on-site field time or hazardous equipment (such as liquid nitrogen). Another study using the bulk-collection method had a higher detection success rate for mammals and birds [30], the difference being that this study captured almost 3.7 times the number of flies. However, the bulk-collection approach differs from most iDNA studies, and there are three major differences between this method and other iDNA techniques: (i) flies are collected in a bait fluid; (ii) they are washed from the bait fluid in which they had been collected; and (iii) flies are extracted using a non-destructive method. Various techniques have been used for capturing flies where individual flies were processed and all have used a destructive protocol for extracting iDNA [19,76]. These studies have reported high detection rates of vertebrate species. Our sampling methodologies may have reduced the overall detection success of the zoo animal species because such a small number of flies were collected and a non-destructive extraction procedure was used. Non-destructive techniques for detecting iDNA signals have been shown to work from arthropod bulk collections that were not necessarily collected for detecting vertebrates [16]. However, using this technique may require much higher volumes of samples to be processed to detect vertebrate signals. The Lynggaard *et al.* [16] study found that only 19.2% of overall samples collected contained non-human vertebrate signals. Therefore, we suggest methodological improvements if using a bulk-collection method for carrion flies. Firstly, traps need to be modified so that flies are not immersed in the collection fluid; and secondly, if fly diversity is not being investigated morphologically, the use of a maceration step for releasing DNA from within flies could also be used.

## 5. Conclusions

We examined the impact of various organism characteristics on vertebrate species detection using iDNA metabarcoding from carrion fly bulk collections in an urban context. We found detections from carrion flies to be spatially localized and context-specific, although we did not have enough detections of zoo vertebrates to find out if taxon and biomass had an effect on vertebrate species detection likelihood. While no monitoring method is unbiased, and detectability between different monitoring methods is variable across taxonomic classes and sizes of animals, understanding these biases is important for interpreting results in an ecological context. We could not study the impact that the number of flies per sample would have on the vertebrate diversity detected, and future studies should explore this question. Also, with such a high diversity of ecology of fly species around the world, it is important to explore these factors in other environments. Therefore, further replication of this study is required to reach a more definitive consensus regarding the non-significant results of both taxonomic group and biomass on vertebrate species detectability. Lessons learned through this study will be important for informing future work using iDNA methodologies in biodiversity monitoring.

## Data Availability

R scripts and the accompanying data are made available [77]. Electronic supplementary material is available online [78].

## References

[1] Legge S , Robinson N , Lindenmayer D , Scheele B , Southwell D , Wintle B . 2018 Monitoring threatened species and ecological communities. Clayton, Australia: CSIRO Publishing.

[2] Balmford A *et al* . 2005 Ecology: the convention on biological diversity’s 2010 target. Science **307** , 212–213. (10.1126/science.1106281) 15653489

[3] Marsh DM , Trenham PC . 2008 Current trends in plant and animal population monitoring. Conserv. Biol. **22** , 647–655. (10.1111/j.1523-1739.2008.00927.x) 18445076

[4] Kouakou CY , Boesch C , Kuehl H . 2009 Estimating chimpanzee population size with nest counts: validating methods in Taï National Park. Am. J. Primatol. **71** , 447–457. (10.1002/ajp.20673) 19235865

[5] Campbell G , Kuehl H , Diarrassouba A , N’Goran PK , Boesch C . 2011 Long-term research sites as refugia for threatened and over-harvested species. Biol. Lett. **7** , 723–726. (10.1098/rsbl.2011.0155) 21450724 PMC3169048

[6] Andersen K , Bird KL , Rasmussen M , Haile J , Breuning-Madsen H , Kjaer KH , Orlando L , Gilbert MTP , Willerslev E . 2012 Meta-barcoding of ‘dirt’ DNA from soil reflects vertebrate biodiversity. Mol. Ecol. **21** , 1966–1979. (10.1111/j.1365-294X.2011.05261.x) 21917035

[7] Ryan E , Bateman P , Fernandes K , van der Heyde M , Nevill P . 2022 eDNA metabarcoding of log hollow sediments and soils highlights the importance of substrate type, frequency of sampling and animal size, for vertebrate species detection. Environ. DNA **4** , 940–953. (10.1002/edn3.306)

[8] McDonald R , Bateman PW , Cooper C , van der Heyde M , Mousavi-Derazmahalleh M , Hedges BA , Guzik MT , Nevill P . 2023 Detection of vertebrates from natural and artificial inland water bodies in a semi-arid habitat using eDNA from filtered, swept, and sediment samples. Ecol. Evol. **13** , e10014. (10.1002/ece3.10014) 37113520 PMC10126312

[9] Rodgers TW , Mock KE . 2015 Drinking water as a source of environmental DNA for the detection of terrestrial wildlife species. Conserv. Genet. Resour. **7** , 693–696. (10.1007/s12686-015-0478-7)

[10] Zhang S , Zhao J , Yao M . 2023 Urban landscape-level biodiversity assessments of aquatic and terrestrial vertebrates by environmental DNA metabarcoding. J. Environ. Manag. **340** , 117971. (10.1016/j.jenvman.2023.117971) 37119629

[11] Newton JP , Bateman PW , Heydenrych MJ , Kestel JH , Dixon KW , Prendergast KS , White NE , Nevill P . 2023 Monitoring the birds and the bees: environmental DNA metabarcoding of flowers detects plant–animal interactions. Environ. DNA **5** , 488–502. (10.1002/edn3.399)

[12] Clare EL , Economou CK , Bennett FJ , Dyer CE , Adams K , McRobie B , Drinkwater R , Littlefair JE . 2022 Measuring biodiversity from DNA in the air. Curr. Biol. **32** , 693–700. (10.1016/j.cub.2021.11.064) 34995488

[13] Lynggaard C , Bertelsen MF , Jensen CV , Johnson MS , Frøslev TG , Olsen MT , Bohmann K . 2022 Airborne environmental DNA for terrestrial vertebrate community monitoring. Curr. Biol. **32** , 701–707. (10.1016/j.cub.2021.12.014) 34995490 PMC8837273

[14] Abrams JF *et al* . 2019 Shifting up a gear with iDNA: from mammal detection events to standardised surveys. J. Appl. Ecol. **56** , 1637–1648. (10.1111/1365-2664.13411)

[15] Calvignac-Spencer S , Leendertz FH , Gilbert MTP , Schubert G . 2013 An invertebrate stomach’s view on vertebrate ecology: certain invertebrates could be used as ‘vertebrate samplers’ and deliver DNA-based information on many aspects of vertebrate ecology. Bioessays **35** , 1004–1013. (10.1002/bies.201300060) 23913504

[16] Lynggaard C , Nielsen M , Santos‐Bay L , Gastauer M , Oliveira G , Bohmann K . 2019 Vertebrate diversity revealed by metabarcoding of bulk arthropod samples from tropical forests. Environ. DNA **1** , 329–341. (10.1002/edn3.34)

[17] Schnell IB , Sollmann R , Calvignac-Spencer S , Siddall ME , Yu DW , Wilting A , Gilbert MTP . 2015 iDNA from terrestrial haematophagous leeches as a wildlife surveying and monitoring tool: prospects, pitfalls and avenues to be developed. Front. Zool. **12** , 24. (10.1186/s12983-015-0115-z) 26430464 PMC4589908

[18] Taberlet P , Coissac E , Pompanon F , Brochmann C , Willerslev E . 2012 Towards next-generation biodiversity assessment using DNA metabarcoding. Mol. Ecol. **21** , 2045–2050. (10.1111/j.1365-294X.2012.05470.x) 22486824

[19] Massey AL *et al* . 2022 Invertebrates for vertebrate biodiversity monitoring: comparisons using three insect taxa as iDNA samplers. Mol. Ecol. Resour. **22** , 962–977. (10.1111/1755-0998.13525) 34601818

[20] Burton AC , Neilson E , Moreira D , Ladle A , Steenweg R , Fisher JT , Bayne E , Boutin S . 2015 Wildlife camera trapping: a review and recommendations for linking surveys to ecological processes. J. Appl. Ecol. **52** , 675–685. (10.1111/1365-2664.12432)

[21] Gogarten JF *et al* . 2020 Fly‐derived DNA and camera traps are complementary tools for assessing mammalian biodiversity. Environ. DNA **2** , 63–76. (10.1002/edn3.46)

[22] Drinkwater R *et al* . 2021 Leech blood-meal invertebrate-derived DNA reveals differences in Bornean mammal diversity across habitats. Mol. Ecol. **30** , 3299–3312. (10.1111/mec.15724) 33171014 PMC8359290

[23] Kocher A , de Thoisy B , Catzeflis F , Valière S , Bañuls AL , Murienne J . 2017 iDNA screening: disease vectors as vertebrate samplers. Mol. Ecol. **26** , 6478–6486. (10.1111/mec.14362) 28926155

[24] Drinkwater R , Williamson J , Clare EL , Chung AYC , Rossiter SJ , Slade E . 2021 Dung beetles as samplers of mammals in Malaysian Borneo—a test of high throughput metabarcoding of iDNA. PeerJ **9** , e11897. (10.7717/peerj.11897) 34447624 PMC8366524

[25] Srivathsan A , Loh RK , Ong EJ , Lee L , Ang Y , Kutty SN , Meier R . 2023 Network analysis with either Illumina or MinION reveals that detecting vertebrate species requires metabarcoding of iDNA from a diverse fly community. Mol. Ecol. **32** , 6418–6435. (10.1111/mec.16767) 36326295

[26] Schnell IB , Thomsen PF , Wilkinson N , Rasmussen M , Jensen LRD , Willerslev E , Bertelsen MF , Gilbert MTP . 2012 Screening mammal biodiversity using DNA from leeches. Curr. Biol. **22** , R262–R263. (10.1016/j.cub.2012.02.058) 22537625

[27] Lee PS , Sing KW , Wilson JJ . 2015 Reading mammal diversity from flies: the persistence period of amplifiable mammal mtDNA in blowfly guts (Chrysomya megacephala) and a new DNA mini-barcode target. PLoS One **10** , e0123871. (10.1371/journal.pone.0123871) 25898278 PMC4405593

[28] Norris KR . 1965 The bionomics of blow flies. Annu. Rev. Entomol. **10** , 47–68. (10.1146/annurev.en.10.010165.000403)

[29] Schubert G , Stockhausen M , Hoffmann C , Merkel K , Vigilant L , Leendertz FH , Calvignac-Spencer S . 2015 Targeted detection of mammalian species using carrion fly-derived DNA. Mol. Ecol. Resour. **15** , 285–294. (10.1111/1755-0998.12306) 25042567

[30] Fernandes K , Bateman PW , Saunders BJ , Bunce M , Bohmann K , Nevill P . 2023 Use of carrion fly iDNA metabarcoding to monitor invasive and native mammals. Conserv. Biol. **37** , e14098. (10.1111/cobi.14098) 37186093

[31] Rodgers TW *et al* . 2017 Carrion fly-derived DNA metabarcoding is an effective tool for mammal surveys: Evidence from a known tropical mammal community. Mol. Ecol. Resour. **17** , e133–e145. (10.1111/1755-0998.12701) 28758342

[32] Bagnall AJ . 2017 Use of iDNA in detecting elusive mammals: a case study on the Indonesian island of Seram. PhD thesis, University of Edinburgh, UK. See https://era.ed.ac.uk/handle/1842/35558

[33] Hoffmann C , Merkel K , Sachse A , Rodríguez P , Leendertz FH , Calvignac-Spencer S . 2018 Blow flies as urban wildlife sensors. Mol. Ecol. Resour. **18** , 502–510. (10.1111/1755-0998.12754) 29328547

[34] Owings CG et al . 2019 Female blow flies as vertebrate resource indicators. Sci. Rep. **9** , 10594. (10.1038/s41598-019-46758-9) 31332240 PMC6646386

[35] Braack LE , Retief PF . 1986 Dispersal, density and habitat preference of the blow-flies Chrysomyia albiceps (Wd.) and Chrysomyia marginalis (Wd.) (Diptera: Calliphoridae). Onderstepoort J. Vet. Res. **53** , 13–18. 3960486

[36] Adams CIM , Hoekstra LA , Muell MR , Janzen FJ . 2019 A brief review of non-avian reptile environmental DNA (eDNA), with a case study of painted turtle (Chrysemys picta) eDNA under field conditions. Diversity **11** , 50. (10.3390/d11040050)

[37] Andruszkiewicz Allan E , Zhang WG , C. Lavery A , F. Govindarajan A . 2021 Environmental DNA shedding and decay rates from diverse animal forms and thermal regimes. Environ. DNA **3** , 492–514. (10.1002/edn3.141)

[38] Rourke ML , Fowler AM , Hughes JM , Broadhurst MK , DiBattista JD , Fielder S , Wilkes Walburn J , Furlan EM . 2022 Environmental DNA (eDNA) as A tool for assessing fish biomass: A review of approaches and future considerations for resource surveys. Environmental DNA **4** , 9–33. (10.1002/edn3.185)

[39] MacLeod J , Donnelly J . 1963 Dispersal and interspersal of blowfly populations. J. Anim. Ecol. **32** , 1. (10.2307/2515)

[40] Spivak M , Conlon D , Bell WJ . 1991 Wind-guided landing and search behavior in fleshflies and blowflies exploiting a resource patch (Diptera: Sarcophagidae, Calliphoridae). Ann. Entomol. Soc. Am. **84** , 447–452. (10.1093/aesa/84.4.447)

[41] Nielsen M , Gilbert MTP , Pape T , Bohmann K . 2019 A simplified DNA extraction protocol for unsorted bulk arthropod samples that maintains exoskeletal integrity. Environmental DNA **1** , 144–154. (10.1002/edn3.16)https://onlinelibrary.wiley.com/toc/26374943/1/2

[42] Riaz T , Shehzad W , Viari A , Pompanon F , Taberlet P , Coissac E . 2011 ecoPrimers: inference of new DNA barcode markers from whole genome sequence analysis. Nucleic Acids Res. **39** , e145. (10.1093/nar/gkr732) 21930509 PMC3241669

[43] Taylor PG . 1996 Reproducibility of ancient DNA sequences from extinct Pleistocene fauna. Mol. Biol. Evol. **13** , 283–285. (10.1093/oxfordjournals.molbev.a025566) 8583902

[44] Bohmann K *et al* . 2022 Strategies for sample labelling and library preparation in DNA metabarcoding studies. Mol. Ecol. Resour. **22** , 1231–1246. (10.1111/1755-0998.13512) 34551203 PMC9293284

[45] Boyer F , Mercier C , Bonin A , Le Bras Y , Taberlet P , Coissac E . 2016 Obitools: a unix-inspired software package for DNA metabarcoding. Mol. Ecol. Resour. **16** , 176–182. (10.1111/1755-0998.12428) 25959493

[46] Callahan BJ , McMurdie PJ , Rosen MJ , Han AW , Johnson AJ , Holmes SP . 2015 Dada2: high resolution sample inference from Illumina amplicon data. Nat. Methods **13** , 581–583. (10.1038/nmeth.3869) PMC4927377 27214047

[47] R Core Team . 2019 R: a language and environment for statistical computing (Version 3.6.1). See https://www.R-project.org/

[48] Mousavi-Derazmahalleh M *et al* . 2021 eDNAFlow, an automated, reproducible and scalable workflow for analysis of environmental DNA sequences exploiting Nextflow and Singularity. Mol. Ecol. Resour. **21** , 1697–1704. (10.1111/1755-0998.13356) 33580619

[49] McMurdie PJ , Holmes S . 2013 phyloseq: an R package for reproducible interactive analysis and graphics of microbiome census data. PLoS ONE **8** , e61217. (10.1371/journal.pone.0061217) 23630581 PMC3632530

[50] QGIS Development Team . 2018 QGIS Geographic Information System (3.2.3-Bonn). See http://qgis.osgeo.org

[51] Hsieh TC , Ma KH , Chao A . 2016 iNEXT: an R package for rarefaction and extrapolation of species diversity (Hill numbers). Methods Ecol. Evol. **7** , 1451–1456. (10.1111/2041-210X.12613)

[52] Chao A , Gotelli NJ , Hsieh TC , Sander EL , Ma KH , Colwell RK , Ellison AM . 2014 Rarefaction and extrapolation with Hill numbers: a framework for sampling and estimation in species diversity studies. Ecol. Monogr. **84** , 45–67. (10.1890/13-0133.1)

[53] Chao A , Ma KH , Hsieh TC . 2016 iNEXT online: software for interpolation and extrapolation of species diversity. See http://chao.stat.nthu.edu.tw/wordpress/software_download/inextonline/

[54] Venables WN , Ripley BD . 2002 Modern applied statistics with S, 4th edn. New York, NY: Springer. (10.1007/978-0-387-21706-2)

[55] Hoffmann C , Stockhausen M , Merkel K , Calvignac-Spencer S , Leendertz FH . 2016 Assessing the feasibility of fly based surveillance of wildlife infectious diseases. Sci. Rep. **6** , 37952. (10.1038/srep37952) 27901062 PMC5128827

[56] Norris KR . 1959 The ecology of sheep blowflies in Australia. In Biogeography and ecology in Australia (eds A Keast , RL Crocker , CS Christian ), pp. 514–544. Dordrecht, The Netherlands: Springer. (10.1007/978-94-017-6295-3)

[57] Perez AE , Haskell NH , Wells JD . 2016 Commonly used intercarcass distances appear to be sufficient to ensure independence of carrion insect succession pattern. Ann. Entomol. Soc. Am. **109** , 72–80. (10.1093/aesa/sav102)

[58] Ji Y *et al* . 2022 Measuring protected-area effectiveness using vertebrate distributions from leech iDNA. Nat. Commun. **13** , 1555. (10.1038/s41467-022-28778-8) 35322033 PMC8943135

[59] Fonseca VG . 2018 Pitfalls in relative abundance estimation using eDNA metabarcoding. Mol. Ecol. Resour. **18** , 923–926. (10.1111/1755-0998.12902)

[60] Murray DC , Coghlan ML , Bunce M . 2015 From benchtop to desktop: important considerations when designing amplicon sequencing workflows. PLoS ONE **10** , e0124671. (10.1371/journal.pone.0124671) 25902146 PMC4406758

[61] Evans MJ , Wallman JF , Barton PS . 2020 Traits reveal ecological strategies driving carrion insect community assembly. Ecol. Entomol. **45** , 966–977. (10.1111/een.12869)

[62] Mas‐Carrió E et al . 2022 Assessing environmental DNA metabarcoding and camera trap surveys as complementary tools for biomonitoring of remote desert water bodies. Environmental DNA **4** , 580–595. (10.1002/edn3.274)

[63] Raemy M , Ursenbacher S . 2018 Detection of the European pond turtle (Emys orbicularis) by environmental DNA: is eDNA adequate for reptiles? Amphib. Reptilia. **39** , 135–143. (10.1163/15685381-17000025)

[64] Regnaut S , Lucas FS , Fumagalli L . 2006 DNA degradation in avian faecal samples and feasibility of non-invasive genetic studies of threatened capercaillie populations. Conserv. Genet. **7** , 449–453. (10.1007/s10592-005-9023-7)

[65] Jones R , Cable J , Bruford MW . 2008 An evaluation of non-invasive sampling for genetic analysis in northern European reptiles. Herpetol. J. **18** , 32–39.

[66] Pearson SK , Tobe SS , Fusco DA , Bull CM , Gardner MG . 2014 Piles of scats for piles of DNA: deriving DNA of lizards from their faeces. Aust. J. Zool. **62** , 507–514. (10.1071/ZO14059)

[67] Ratsch R , Kingsbury BA , Jordan MA . 2020 Exploration of environmental DNA (eDNA) to detect Kirtland’s snake (Clonophis kirtlandii). Animals **10** , 1057. (10.3390/ani10061057) 32575432 PMC7341209

[68] Wilting A *et al* . 2022 Creating genetic reference datasets: indirect sampling of target species using terrestrial leeches as sample ‘collectors’. Environ. DNA **4** , 311–325. (10.1002/edn3.256)

[69] Singer GAC , Fahner NA , Barnes JG , McCarthy A , Hajibabaei M . 2019 Comprehensive biodiversity analysis via ultra-deep patterned flow cell technology: a case study of eDNA metabarcoding seawater. Sci. Rep. **9** , 5991. (10.1038/s41598-019-42455-9) 30979963 PMC6461652

[70] Rojahn J , Gleeson DM , Furlan E , Haeusler T , Bylemans J . 2021 Improving the detection of rare native fish species in environmental DNA metabarcoding surveys. Aquat. Conserv. **31** , 990–997. (10.1002/aqc.3514)

[71] Shehzad W , Riaz T , Nawaz MA , Miquel C , Poillot C , Shah SA , Pompanon F , Coissac E , Taberlet P . 2012 Carnivore diet analysis based on next-generation sequencing: application to the leopard cat (Prionailurus bengalensis) in Pakistan. Mol. Ecol. **21** , 1951–1965. (10.1111/j.1365-294X.2011.05424.x) 22250784

[72] Vestheim H , Jarman SN . 2008 Blocking primers to enhance PCR amplification of rare sequences in mixed samples: a case study on prey DNA in Antarctic krill stomachs. Front. Zool. **5** , 12. (10.1186/1742-9994-5-12) 18638418 PMC2517594

[73] Carvalho CS , de Oliveira ME , Rodriguez-Castro KG , Saranholi BH , Galetti PM . 2022 Efficiency of eDNA and iDNA in assessing vertebrate diversity and its abundance. Mol. Ecol. Resour. **22** , 1262–1273. (10.1111/1755-0998.13543) 34724330

[74] Benbow EM , Tomberlin JK , Tarone AM . 2015 Carrion ecology, evolution, and their applications. Boca Raton, FL: CRC Press.

[75] Bogoni JA , Hernández MIM . 2014 Attractiveness of native mammal’s feces of different trophic guilds to dung beetles (Coleoptera: Scarabaeinae). J. Insect Sci. **14** , 299. (10.1093/jisesa/ieu161) 25528749 PMC5657881

[76] Calvignac-Spencer S , Merkel K , Kutzner N , Kühl H , Boesch C , Kappeler PM , Metzger S , Schubert G , Leendertz FH . 2013 Carrion fly-derived DNA as a tool for comprehensive and cost-effective assessment of mammalian biodiversity. Mol. Ecol. **22** , 915–924. (10.1111/mec.12183) 23298293

[77] Fernandes K . 2024 Data from: Zoo Flies—R Scripts and Data. Zenodo. (10.5281/zenodo.10570058)

[78] Fernandes K , Bateman PW , Saunders BJ , Gibberd M , Bunce M , Bohmann K , et al . 2024 . Data from: Analysing the effects of distance, taxon, and biomass on vertebrate detections using bulk-collected carrion fly iDNA. Figshare. (10.6084/m9.figshare.c.7124929) PMC10987983 38577218

